# Health Tract No. 203

**Published:** 1864-04

**Authors:** 


HEALTH TRACT No. 203.

### HOUSEHOLD KNOWLEDGE.

Windows are kept free from ice by painting the glass with alcohol with a brush or sponge.

Odors from boiling ham, cabbage, etc., are prevented by throwing red pepper-pods or a few pieces of charcoal into the pot.

Percussion-caps are found to poison children, if swallowed.

Pigeons are hatched in eighteen days; chickens, twenty-one; turkeys, twenty-six; ducks and geese, thirty.

A cement which is a good protection against weather, water, and fire, to a certain extent, is made by mixing a gallon of water with two gallons of brine, then stir in two and a half pounds of brown sugar and three pounds of common salt; put it on with a brush like paint.

Eggs, for cooking purposes.—One table-spoon of corn-starch is said to be equal to one egg.

French Rolls.—Add two ounces of butter and a little salt to a pint of boiled milk; while tepid, sift in one pound of flour, one beaten egg, one tablespoon of yeast; beat these altogether well; when risen, form the rolls with as little handling as possible; bake on tins.

Boiling Potatoes.—Put potatoes of equal size into water while boiling; when done, pour off the water, scatter in some salt, cover the pot with a coarse cloth, and return it to the fire for five minutes, when they are ready for the table; even watery potatoes are thus made mealy.

Common cut-nails are easily driven into hard wood if rubbed with a little soft-soap; the saliva is better than nothing for that purpose.

Never condemn your neighbor unheard; there are always two ways of telling a story.

Potatoes.—The best way to cook a potato is to bake or roast it in an oven; when done, crack the skins open and allow them to dry out for a few minutes before placing them on the table.

Quarrels.—To avoid family quarrels, let the quarreling wretch have it all to himself; reply never a word.

Corns, *new cure!*—Let a piece of pure India-rubber, the twentieth of an inch thick, remain in constant contact with the corn, which should be kept closely and well pared; it requires four or five weeks.

Cider Vinegar.—Take the water in which dried apples have been soaked and wash, strain it well, add a pound of sugar.

